# Optical Fiber Embedded in Epoxy Glass Unidirectional Fiber Composite System

**DOI:** 10.3390/ma7010044

**Published:** 2013-12-20

**Authors:** Irina Severin, Rochdi El Abdi, Guillaume Corvec, Mihai Caramihai

**Affiliations:** 1 Department Materials Technology/Computer Science, Politehnica University of Bucharest, 313 Splaiul Independentei, Bucharest 060042, Romania; E-Mail: m.caramihai@ieee.com; 2 Larmaur, ERL CNRS 6274, University of Rennes 1, Beaulieu Campus, Rennes Cedex 35042, France; E-Mails: relabdi@univ-rennes1.fr (R.E.A.); guillaume.corvec@gmail.com (G.C.)

**Keywords:** optical fiber, epoxy/E-glass composite, manufacturing, interface adhesion, pull-out test, Weibull plots, MEB

## Abstract

We aimed to embed silica optical fibers in composites (epoxy vinyl ester matrix reinforced with E-glass unidirectional fibers in mass fraction of 60%) in order to further monitor the robustness of civil engineering structures (such as bridges). A simple system was implemented using two different silica optical fibers (F1—double coating of 172 μm diameter and F2—single coating of 101.8 μm diameter respectively). The optical fibers were dynamically tensile tested and Weibull plots were traced. Interfacial adhesion stress was determined using pull-out test and stress values were correlated to fracture mechanisms based on SEM observations. In the case of the optical fiber (OF) (F1)/resin system and OF (F1)/composite system, poor adhesion was reported that may be correlated to interface fracture at silica core level. Relevant applicable results were determined for OF (F2)/composite system.

## 1. Introduction

Composite materials are now becoming accepted for use in many major structural applications, particularly in the space, aerospace, marine, civil engineering, and automotive industries. These materials with their high specific strength and stiffness are gradually replacing their metal counterparts due to reduced weight, improved wear and corrosion resistance, increased fatigue life, and the ability of the material and component to be formed at the same time. The wide ranges of structural composites are based on polymeric matrices with glass or carbon reinforcement [1].

Optical fiber sensors have gained much attention in recent years for a variety of physical and chemical measurements. Intense research has been performed to develop “special fibers” for integration in smart structures in order to replace conventional sensors [2–4] and to provide information on the continuous evolution of damaged structures under mechanical stress, thus monitoring structural robustness [5,6].

The efficiency of optical fiber sensors to continuously monitor structure deformation and reinforcement cracks depends mainly on strength transfer at the interface level between the embedded optical fiber and the composite structure [7,8]. In order to assess mechanical properties at interface level and composites performance, certain testing, such as the pull-out test, has been developed [9,10]. The test may be used to reveal the bonding quality between the optical fiber sensor and the composite structure. The interfacial adhesion strength τ*_d_* appears as a critical factor for structural robustness monitoring being given by:

$$\tau_{d}=F_{d}/\text{2}\cdot \pi \cdot R\cdot L_{e}$$ (1)

where *F_d_* is the maximum force corresponding to the linear zone of the force *versus* displacement curve; *R* is the core optical fiber radius and *L_e_* the optical fiber embedded length.

In the context of recently developed optical fibers for distributed sensors and other smart structures, the reliability of optical fibers appears as a critical factor. Testing procedures and damage mechanisms identified on a series of experimental studies are given elsewhere [11].

The present study has the aim to manage a reliable fabrication process of embedding optical fiber in an epoxy/glass fiber composite and to obtain basic data on a simple system. In a further step, Bragg grating optical fiber is envisaged to be embedded in a similar composite structure in order to implement a smart structure sensitive to flexion stress.

## 2. Experimental Procedure

### 2.1. Composite Fabrication

The polymer matrix used in this study, provided by the French company DFC-DCP Pultrusion (Creil, France), is a mixture of epoxy vinylester resin DERAKANE 470-36 and a catalytic system composed of Styrene, Perkadox 16 and Trigonox C. After homogenously stirring the epoxy resin, the powdered Perkadox 16 is firstly diluted in Styrene so as to form a homogeneous Styrene/Perkadox 16 system, and then the Trigonox C is added. For the experimental implementation, small matrix quantities were prepared, such as 30 g of epoxy resin and 0.66 g Styrene, 0.264 g Perkadox 16 and 0.108 g Trigonox C.

Composite samples were prepared, reinforcing the epoxy matrix with E-glass fibers Roving 4800 Tex having a density of 2.62 g/cm^3^. A mass fraction of 63% glass fiber in epoxy matrix was envisaged, meaning an incorporation of 12 roving in the implemented composite sample. To calculate the roving number, the following relation is used:

$$N=\;0.\text{545833}f\cdot V_{com}$$ (2)

where *f* is glass fiber volume fraction and *V_com_* the composite volume. Roving 4800 Tex means 1 roving = 0.0048 g/mm. For the samples of 50 mm × 5 mm × 10 mm, 12 roving correspond to a volume fraction of 40% and a mass fraction of 63%, respectively. The composite samples were weighted after fabrication and effective mass fraction ranging between 55% and 65% were obtained.

In order to characterize the interfacial adhesion between the optical fiber and the epoxy vinylester/glass fiber composite material (OF/C), a simple system composed of a single optical fiber embedded in epoxy vinylester resin (OF/R) was preliminarily studied. The system is a unidirectional composite, where glass fibers have the sample length direction and the optical fiber is centrally embedded in the reinforcement direction.

Two different optical fibers elaborated by iX Fibers S.A.S. company (Lannion, France) were used: firstly, the fiber, denoted by F1, having a 80 μm silica core and two acrylate polymer layers of 172 μm secondary coating diameter and secondly, the fiber, denoted by F2, having a 80 μm silica core, but a single acrylate polymer layer of 101.8 μm fiber coating diameter.

As optical fiber surface has determined fracture to a large extent, external coating appears critical. This coating is polymeric in most cases, and modern optical fibers are coated by two different layers: a soft coating at glass surface and a hard coating at external surface. The coating makes a protection against scratches that occur in normal handling and it reduces water activity at glass surfaces. Polymeric coatings (e.g., epoxy-acrylate) are preferred in practice because they are more efficient at inhibiting surface defects. As one will notice later in our study, regarding MEB examinations (see Section 3.3), fiber F1 has the typical section with two layers, but fiber F2 was subjected to a thermal treatment and presents one layer surrounding the silica core.

The thermal treatment consisted of exposure at 200 °C curing temperature for 100–200 h. The fiber behavior appears similar to polyimide, those polymers containing chemical groups that promote solubility without affecting significantly mechanical properties. Moreover, curing at 200 °C for 100 h, leads to the lost of 1.5% of the mechanical strength as compared to 9% lost in the case of polyimide fiber. Fiber parameters are given in Table 1.

**Figure 1.**
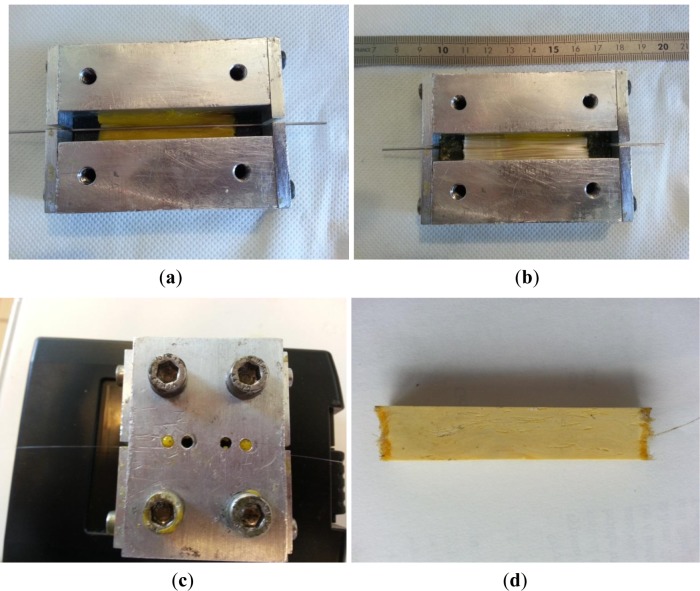
Composite manufacturing experimental procedure. (**a**) Pre-impregnated E-glass roving; and optical fiber steel guide placement in the mold (**b**) mold fill-in; (**c**) mold closure—see optical fiber ends and resin surplus evacuation system and (**d**) composite sample.

The sample preparation consisted of incorporating the glass fiber in the resin into a mold and embedding the optical fiber centrally positioned into the sample. As seen in Figure 1, the glass roving is placed in the mold and, superimposed layer by layer (6 roving), is carefully impregnated with the resin until half the mold is filled, and then a fine steel cylinder guide is placed on top in order to allow optical fiber insertion. The mold is filled in, as previously described (other 6 roving), and then the optical fiber is gently inserted in the fine guide. Before closing the mold with a controlled screw torque of 2 Nm, the steel guide is gently and carefully extracted. For safety reasons, all operations were performed into an extractor hood.

Then, curing in preheated oven at 80 °C temperature for 90 min was applied and samples were extracted after 60–90 min.

### 2.2. Pull-Out Testing

**Figure 2.**
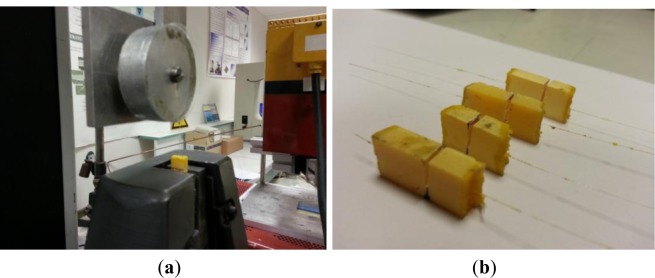
(**a**) Pull-out testing; and (**b**) sample prepared for pull-out tests.

Following the procedure, series of F1 optical fiber embedded in resin and in composite, respectively and F2 optical fiber embedded in composite were prepared in order to perform pull-out tests. Using a LLOYD LR 50K tensile testing setup (Lloyd Instruments Ltd., Fareham, Hampshire, UK), a gripping force was applied, as seen in Figure 2a. The optical fiber end is rolled up on a pulley of 50 mm in diameter and covered with a powerful adhesive so as to prevent fiber slip during testing. When the optical fiber was pulled-out, the fiber breakage occurred at the bit. Thus, a notch was introduced into the specimen as seen in Figure 2b, at a distance of 8 mm from the sample end. The cross head speed was set at 1 mm·min^−1^, which corresponds to a strain rate of about 0.02 min^−1^. The de-bonding force F was considered as the maximum force preceding partial de-bonding. A scanning electron microscope (SEM) (Field Emission Scanning Electron Microscope-type JEOL JSM 6301F, Tokyo, Japan) was used to investigate the surface of optical fibers after pull-out tests.

### 2.3. Optical Fiber Dynamic Tensile Testing

A tensile bench Lloyds Instruments LR 50K (maximum: 100 N) was used. In order to be dynamically tensile tested, sample fibers were three tours rolled on the cylinder pulley having 50 mm in diameter. The pulleys were covered with a powerful double face adhesive; the mechanical properties of the adhesive layer appeared to be important controlling factors. Despite the standard conditions, for economy and testing time reasons, sample testing free length was chosen to 200 mm. A testing strain rate of 20, 100, 200, respectively 500 mm/min was chosen. These rates were selected in order to correspond to 10, 50, 100, respectively 250%/min as compared to sample main length. In the case of the reference fiber, at least 20 samples were tested and Weibull plots were traced, then *n_d_*-stress (*n_d_*, dynamic stress corrosion parameter) corrosion factor was calculated.

In order to briefly evidence several parameter influences (oven treatment for polymerization or catalytic system—Trigonox C) on optical fiber mechanical properties, series of F1 fiber were treated, then tensile tested and compared to as-received fiber.

Results treatment is then usually based on Weibull plots, despite some opponents concerning the adequacy of Weibull distribution use in case of tensile experiments [12–14]. Even if at least 50 samples are proposed for a reasonable estimation using Weibull, due to our mainly aim of comparing fiber mechanical strength subjected to different etching environments, the 20 series used for Weibull treatment appeared relevant.

## 3. Experimental Results and Discussion

### 3.1. Optical Fiber Characterization

Based on our previous experimental studies, the optical fiber was dynamically tensile tested for four different strain rates of 0.1, 0.5, 1 and 2.5 min^−1^ corresponding respectively to the tensile testing cross speed of 20, 100, 200 and 500 mm/min. For each tested sample, we determined the stress to fracture, and then the results were treated through a statistical approach using the Weibull theory. The classical Weibull plots of the logarithm function of the cumulative failure probability (*F_k_*) related to the logarithm of the stress to fracture (*C*) has allowed us to calculate and to compare the statistical parameters and the *n_d_*-stress corrosion parameter.

It is assumed that fracture at the most critical flaw on a fiber leads to total failure. For brittle materials as silica optical fibers, strength results obtained from tensile tests present a significant scattering. Then, the statistical Weibull method is commonly used. This method leads us to obtain the mean stress value (strength at 50% fracture probability of the Weibull plot), the medium stress, the Weibull slope and the distribution of the critical flaw size in the sample. The statistical Weibull law gives a relationship between the probability *F_K_* of fiber fracture and the applied stress (*C*). The evolution of ln[−ln(1 − *F_K_*)] according to ln*C* is called Weibull diagram.

The slope *p* of the curve ln*C* *versus* ln (where is the testing rate in μm/s) is related to the dynamic stress corrosion parameter *n_d_*, a parameter characterizing the material capacity to resist to a stress. The accepted stress corrosion parameter is ~20 for high strength fiber.

**Figure 3.**
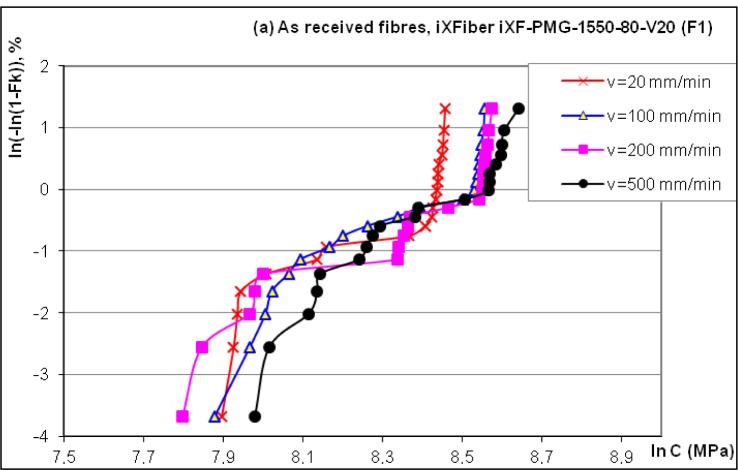

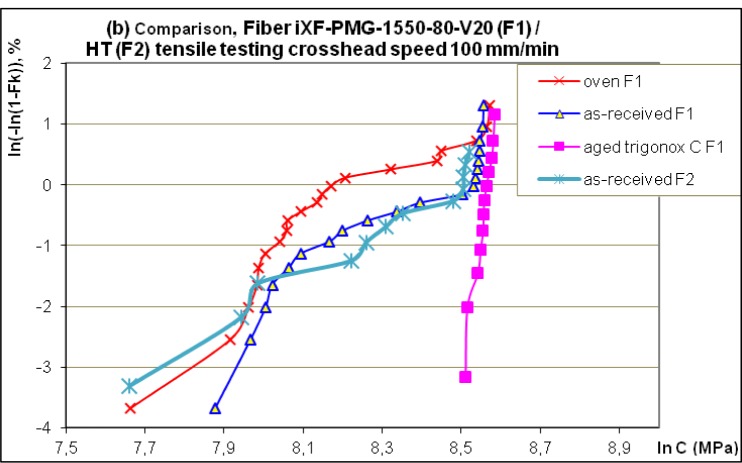
(**a**) Weibull plots for reference fiber F1 (double polymer coating) and (**b**) comparison of different etching factors tensile strength (F2—single polymer coating).

Weibull plots corresponding to the fiber denoted by F1 (two polymer layer coating to protect the silica core) are given in Figure 3a.

The medium values for the tensile stress correlated to the given strain rates were respectively 4034.3, 4183.7, 4328.5 and 4426.6 MPa. As compared to our previous dynamic tensile testing performed on Verrillon Inc. (North Grafton, MA, USA) and Alcatel optical fibers, one may conclude that F1 fiber presents quite broad dispersion and lower tensile stress. The stress corrosion factor *n_d_* = 33.8 appears adequate with a linear interpolation of *R^2^* = 0.98.

Due to experimental reasons, the F2 fiber (single polymer layer coating) was tested for one speed (100 mm/min) and quite similar results are obtained, as seen in Figure 3b, with a medium stress of 4179.4 MPa, as compared to 4183.7 MPa for F1. The same broad dispersion and low tensile stress are reported.

The fabrication parameters influence the optical fiber tensile strength as seen in Figure 3b, thus heating in oven for 90 min at 80 °C led, as expected, to strength decrease, but the Trigonox C (resin compound) determined the strength increase with a more coherent and steeper distribution. Several testing performed with fiber F1 plunged for 7 days in water at room temperature (20 °C) confirmed our previous observations concerning slight increase of strength, but limited dispersion.

### 3.2. Pull-Out Testing*—*Interface Adhesion Characterization

The fabricated systems OF (F1)/Resin and OF (F1, F2)/Composite were prepared as described. The notch at 8 mm length from the sample end was cut to control the optical fiber embedding length *L*_e_. The force (in N) *versus* displacement (in mm) curve was recorded and the maximum value was considered for comparison.

The debonding force *F*_d_ was taken as the maximum force preceding partial debonding.

**Figure 4.**
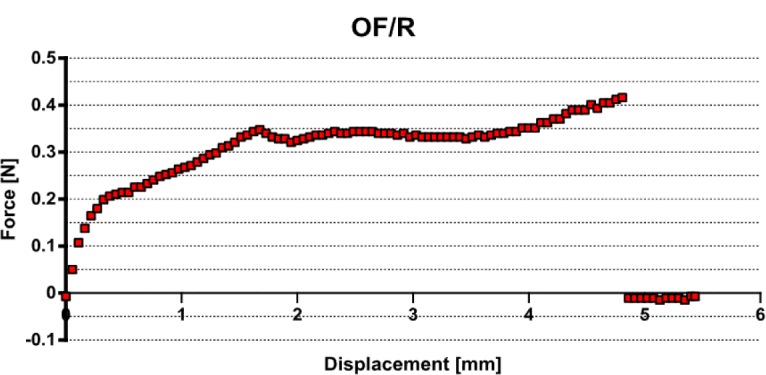
Pull-out record for optical fiber F1 embedded in epoxy-vinyl resin.

In the case of optical fibers embedded in resin, noted OF (F1)/Resin, a poor interface adhesion is reported for all samples with a maximum of 0.98 N. Using the linear model Equation (1), an interfacial adhesion stress of 0.46 MPa is reported. The pull-out curves are similar to Figure 4.

**Figure 5.**
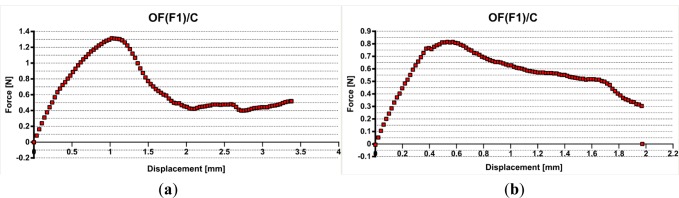
Pull-out record for optical fiber F1 embedded in epoxy composite/glass fiber. (**a**) Maximum 1.32 N and (**b**) maximum 0.8 N.

The optical fiber F1 embedded in the composite presented poor adhesion interface, too, slightly higher than the resin, but still low. A maximum value of 1.32 N (see Figure 5a) was recorded that corresponds to an interfacial adhesion stress of 0.66 MPa. but in the most cases debonding forces of 0.8–0.9 N were registered (see Figure 5b), leading to similar stresses as for non-reinforced matrix case.

**Figure 6.**
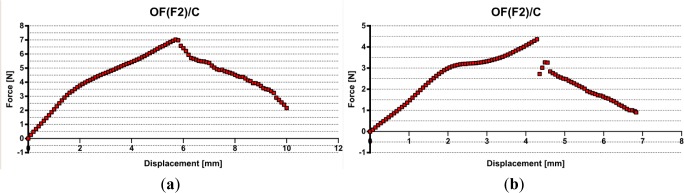
Pull-out record for optical fiber F2 embedded in epoxy composite/glass fiber. (**a**) Maximum 7.1 N and (**b**) maximum 3.5 N.

Finally, the optical fiber F2 embedded in the composite presented better adhesion at OF/composite interface (Figure 6).

A maximum value of 7.1 N (see Figure 6a) was recorded that corresponds to an interfacial adhesion stress of 3.53 MPa and several stick-slip phenomena were noticed (see Figure 6b), as previously reported [15], but for lower debonding forces.

The decrease of the force seems to be controlled by friction over the entire embedded length. The composite rigidity is higher than that of the non-reinforced resin. On the other hand, for the composite sample reinforced with glass fibers, one may expect less friction at the interface between the optical fiber and the composite than for non-reinforced resin in contact with the optical fiber coating. Thus, in the case of the composite sample with glass fibers, the interfacial debonding can propagate with a small instability and the obtained curve appears quasi-continual [15].

### 3.3. SEM Observations—Interface Adhesion Characterization

**Figure 7.**
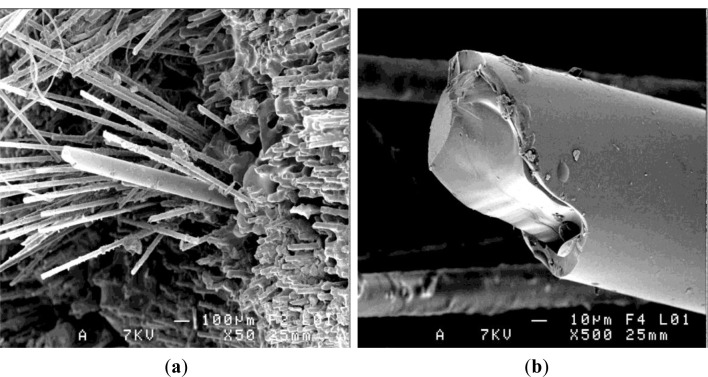
(**a**) Micrograph of the optical fiber F2 embedded in the glass reinforced vinyl-ester composite; (**b**) detail of the optical fiber (see core and single polymer layer).

**Figure 8.**
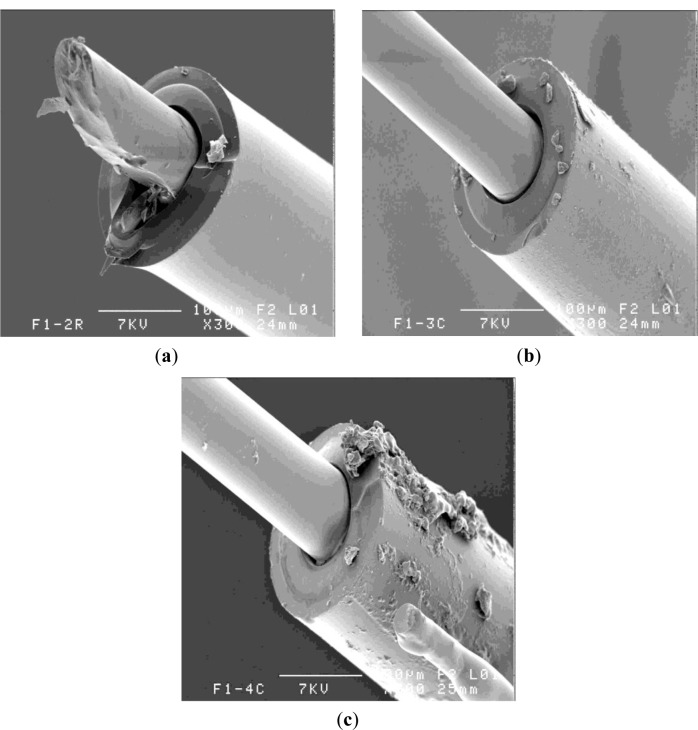
Interface fracture in F1 fiber embedded in (**a**) resin and in (**b**,**c**) composite.

An overall image of the composite end is given in Figure 7 with the detail of the optical fiber. The single polymer coating may be seen in this case of F2 optical fiber centrally embedded in the composite, as compared to the double polymer coating in the case of F1 optical fiber (Figure 8a).

In the case of the optical fiber (F1—double polymer coating) embedded in the resin, Figure 8a, examined after the pull-out test, the external polymer coating presented few resin matrix traces, but following the polymerization process the silica core interface appeared detached.

The physico-chemical nature of interfacial bonds depends on both elaboration temperature, and the time maintained at this elaboration temperature. The kinetics of interfacial bonding creation can be connected to a diffusive mechanism. The curing time enhances the formation of chemical bonds, and improves the diffusion mechanism at the interface optical fiber/resin. The adhesion at the interface results from the interdiffusion of macromolecular chains between the two polymer surfaces (resin and coating), thus forming an interphase. This interdiffusion could originate from a good compatibility between the acrylate coating and the vinyl ester resin, (both have hydroxyl groups, able to ensure a good wettability) or it could be the result of a macromolecular mobility, which leads to a tangle by reptation [15,16].

At the silica/polymer coating interface the coherence appears affected, as seen in Figure 8b, and this polymer detachment around the silica seems to be responsible for the low interfacial adhesion stress in case of resin and composite embedding F1 fiber. As noted, the resin filaments on the optical fiber external coating appeared more frequent in the composite case, Figure 8c.

**Figure 9.**
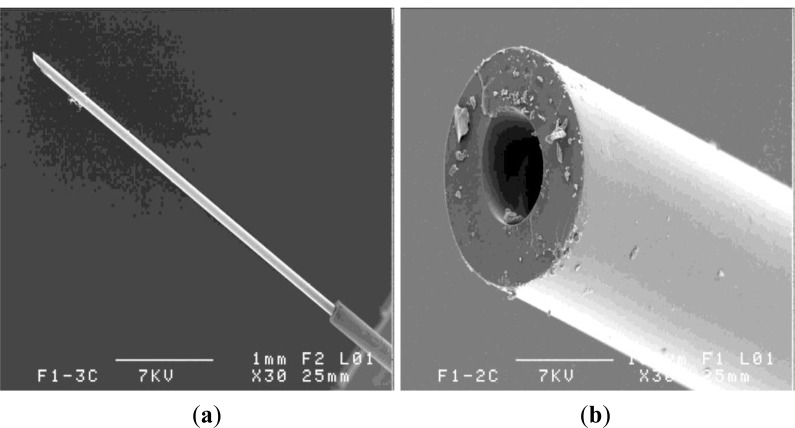
Micrograph of F1 fiber embedded in composite. (**a**) Core stripping; and (**b**) core damage.

Despite attentive precautions and rigorous fabrication among composite prepared samples, one may conclude that, following the pull-out test, half of the samples presented as-stripped optical fiber core (Figure 9a) or polymer coating with silica core. Losses because of detached coatings were due to core fracture and damaged adherence of inner soft polymer coating (Figure 9b).

**Figure 10.**
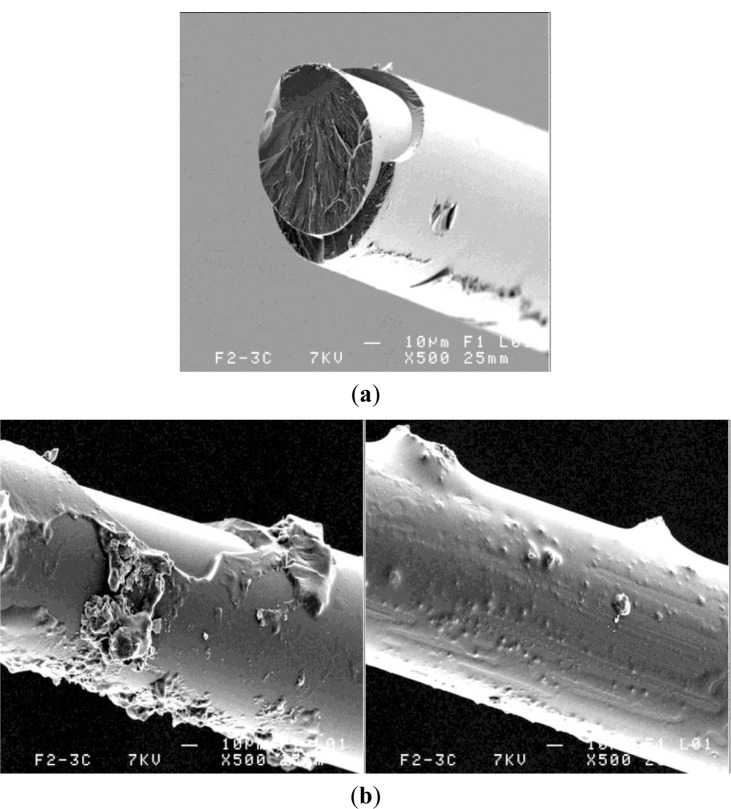

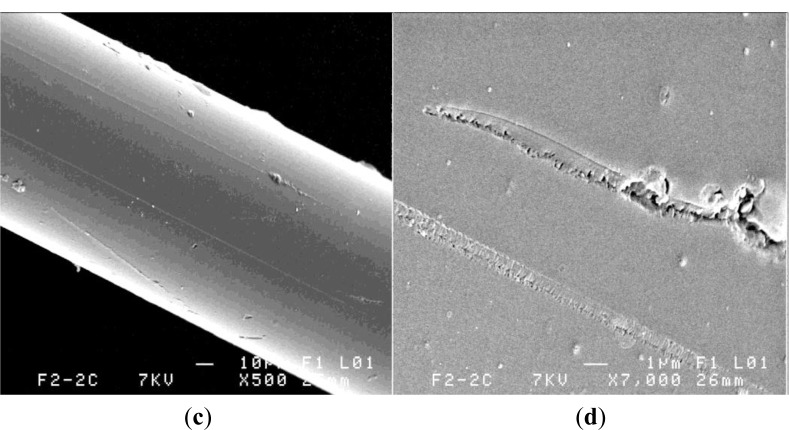
Micrograph of F2 fiber embedded in composite presents: (**a**) resin filaments; (**b**) polymer fracture, polymer detachment and glass reinforcements imprints; (**c**) scratches and (**d**) zoom of scratches.

For the F2 fiber embedded in the composite that has led to improved adhesion of the optical fiber, one may notice good cohesion at the silica core/polymer coating interface, see Figure 10a.

In certain zones, maybe due to stress concentration, polymer fracture and detachment along the optical fiber (Figure 10b) was noticed, as were frequent resin traces (filaments and drops), sometimes oriented in the reinforcement direction.

Glass fiber reinforcements may scratch the polymer coating and certain imprints are also visible (Figure 10c), but this observation was not as frequent as previously reported [17].

SEM observations have confirmed that polymer detachment were more rare in the F2 fiber case than in the case of F1 fiber embedded similarly in the composite system.

## 4. Conclusions

As-received optical fibers F1 (double coating 172 μm diameter) and F2 (single coating 101.8 μm diameter) present similar mechanical strength with medium tensile strength between 4 and 4.4 GPa with broad dispersion and adequate stress corrosion factor (*n_d_* = 33.8). Based on previous experimental studies, polymeric coatings are efficient to inhibit surface defects at the silica core interface.

The main difference between the investigated fibers is the thermal treatment applied in the case of F2 (single coating) that leads to a behavior close to polyimide.

As SEM observations exhibited, in the case of optical fiber with double layer polymer coating (F1), embedded in resin or composite, the soft polymer layer around the silica core retracted, probably due to the internal stresses following polymerization cycle of the resin matrix. Therefore, the interface has lost its coherence, leading to low interfacial adhesion stresses ranging from 0.4 to 0.66 MPa.

Optical fibers heat treatment at curing parameters revealed a strength decrease, but the resin catalytic system (Trigonox C) appeared favorable; the strength slightly increased and presented steeper distribution. Water at environment temperature has a similar effect after one week of exposure of the optical fiber, observations that appeared coherent to our previous results on other silica optical fibers

One may conclude that fabrication parameters acted synergistically on the optical fiber interfacial adhesion in the resin matrix, but certain precautions and a rigorous procedure should be followed to manage a coherent interface. Glass reinforcements of 55%–65%, fraction in mass, acted in the favor of the interfacial strength, the composite case exhibiting slightly better interfacial adhesion stress than the non-reinforced matrix resin, which may be explained through the harmful effect of internal stresses concentration in composite systems.

Finally, the optical fiber with a single layer coating (F2) subjected to a thermal treatment embedded in the composite system has allowed better quality at interface level. The polymer coated fiber appeared to be detached in certain zones following the pull-out test, scratched by reinforcements on its external surface and resin filaments. Drops were seen too, but the silica core/polymer coating revealed coherence and the interfacial adhesion stress is reported at 3.5 MPa.

Our objective to manage a reliable fabrication process of embedding optical fiber in an epoxy/glass fiber composite has been attainted and subsequent testing of Bragg gratings optical fiber (using the F2 single coating) should follow.

## Figures and Tables

**Table 1.** iX Fiber references.

Parameter	F1	F2
Optical fiber reference	iXF-PMG-1550-80-V20	iXF-PMG-1550-80-HT
Attenuation@1550 nm, dB/km	1.15	1.68
Cutoff wavelength, nm	1300	1318
Bare fiber/coating diameter, μm	80/172	80/101.8
